# Outcomes in Oxacillinases β-Lactamases (OXA-48) and New Delhi Metallo-β-Lactamase (NDM-1)-Producing, Carbapenem-Resistant Klebsiella Pneumoniae Isolates Obtained From Bloodstream Infections

**DOI:** 10.7759/cureus.27197

**Published:** 2022-07-24

**Authors:** Anuragani Verma, Parul Jain, Piyush Tripathi, Raj K Kalyan, Sheetal Verma, Vimala Venkatesh

**Affiliations:** 1 Microbiology, King George's Medical University, Lucknow, IND; 2 Clinical Microbiology, King George's Medical University, Lucknow, IND

**Keywords:** mortality, infection control, colistin, carbapenemase genes, antibiotic resistance

## Abstract

Background: Carbapenemase-producing *Klebsiella pneumoniae* (CRKP) has become a menace in several intensive care units, which needs to be controlled immediately after being reported by a laboratory. Detection in the laboratory is usually done using phenotypic methods and it is not known whether knowledge of these genes helps in individual patient management. This study aimed to compare the outcomes of oxacillinases β-lactamases (OXA-48) and New Delhi metallo-β-lactamase (NDM-1)-producing CRKP isolates, the two most common carbapenemases reported from India, obtained from patients with bloodstream infections in an ICU in a tertiary care center in North India and to compare the different laboratory methods for their detection.

Materials and methods: *Klebsiella pneumoniae* isolates obtained from the blood culture of patients admitted to various ICUs were subjected to conventional polymerase chain reaction (PCRs) for blaNDM and blaOXA48-like genes. Those positive for any of the genes were tested by the modified carbapenem inactivation method (mCIM) and if found positive were also subjected to ethylenediamine tetraacetic acid (EDTA)-modified carbapenem inactivation method (eCIM). Antibiotic susceptibility tests (AST) were performed and clinical data were recorded.

Results: A total of 49 isolates were positive for one or more carbapenemase genes (30 {61.2%} for blaNDM gene only, 13 {26.5%)} for blaOXA48-like gene only, and six {12.2%} for both). The sensitivity, specificity, positive predictive value (PPV), and negative predictive value (NPV) of mCIM were found to be 77.6%, 100%, 100%, and 78.9%, respectively. Statistically significant differences were found in the AST pattern between the isolates with two genes. Increased MIC levels of colistin were observed, though they lay in the sensitive range. Mortality occurred in all six patients who were infected with CRKP harboring both the genes though no significant difference was observed in NDM and OXA-48 producing CRKP isolates.

Conclusion: Surveillance of carbapenemase genes in a hospital setting is essential. The possible reasons for the low diagnostic accuracy of mCIM and differences in AST patterns are discussed.

## Introduction

*Klebsiella pneumoniae* is an important cause of healthcare-associated infections. It accounts for about 8% of all nosocomial infections and for up to 14% of cases of primary bacteremia [1,2]. The emergence and dissemination of drug resistance in *K. pneumoniae* strains have made the situation even worse. It has been observed that up to 80% of all *K. pneumoniae* strains from India are resistant to cephalosporins and up to 60% are carbapenem-resistant (CRKP) besides the beta-lactams (including carbapenems), they are also resistant to several non-beta-lactam antibiotics. Due to the limited therapeutic options available, mortality rates of as high as 40-50% have been reported in patients infected with CRKP [3].

Carbapenem resistance is primarily mediated by the production of carbapenemases (carbapenem hydrolyzing -lactamases), which have been found in *K. pneumoniae* isolates to fall into three categories: (1) metallo-lactamases or molecular class B lactamases (New Delhi metallo-lactamase/New Delhi metallo-β-lactamase {NDM-1}, IMP, VIM) that hydrolyze all lactams except monobactams and are inhibited by ethylenediamine tetraacetic acid (EDTA) but not clavulanic acid; (2) serine - lactamases of molecular class A (NMC/IMI, SME, KPC, and GES) that hydrolyze even monobactams are inhibited by clavulanic acid and tazobactam but not by EDTA; (3) molecular class D serine β-lactamases (oxacillinases β-lactamases {OXA-48}) that do not hydrolyze monobactams and are poorly inhibited by clavulanic acid and EDTA [4]. These properties are used for differentiation in the laboratory. Most of these carbapenemases are acquired either by mutation or by horizontal gene transfer.

Among these classes of carbapenemases, NDM-1 (produced by blaNDM gene) and OXA-48 (produced by blaOXA48-like gene) have been reported to be the commonest in India [5]. Phenotypic tests are presently being used in many bacteriology laboratories to differentiate between these genes. But the efficiency of these tests in routine laboratory settings and the impact of differentiating these genes on patient outcomes are sparsely known. Therefore, the present study aimed to compare the outcomes in terms of mortality and susceptibility to antibiotics of OXA-48 and NDM-1-producing carbapenem-resistant *K. pneumoniae* isolates obtained from patients with bloodstream infections in an ICU in a tertiary care center in North India and to compare the different laboratory methods used for their detection.

## Materials and methods

All *K. pneumoniae* isolates obtained from the blood culture of patients admitted in various ICUs of King George's Medical University, Lucknow, India, between August 2018 and July 2019 were included. The isolates were characterized using conventional biochemicals and VITEK 2 (Biomerieux: Marcy-IEtoile, France) compact system. Genomic DNA was extracted from these isolates by the boiling method as described previously [6]. Conventional PCRs were done to detect blaNDM [7] and blaOXA48-like genes [8]. The primer sequences used and cycling conditions are mentioned in Table 1. The polymerase chain reaction (PCR) was done in a final reaction volume of 20μl containing 10μl of 2X universal PCR master mix (Thermo Fisher Scientific PCR master mix: Vilnius, Lithuania), 2μl of 10μM of each primer, 4μl of nuclease-free water, and 4μl of DNA template. After amplification, all bands were subjected to electrophoresis in 1.5% agarose gel and detected with ethidium bromide staining under UV illumination. Band sizes of 800bp and 948 bp were considered as positive for blaNDM and blaOXA48-like genes, respectively, and isolate harboring them were called CRKP isolates (Table 1).

**Table 1:** Primer sequences and cycling conditions of conventional PCRs for blaNDM and blaOXA48-like genes PCR: polymerase chain reaction; OXA-48: oxacillinases β-lactamases; NDM: New Delhi metallo-β-lactamase

Primer name	Primer sequence	Product size	Thermal cycling conditions	
OXA-48-F	5’-TATATTGCATTAAGCAAGGG-3’	800bp	(1) Enzyme activation; (2) 30 cycles of initial denaturation annealing extension; (3) final extension	95°C for 15 min, 94 °C for 30s, 59°C for 1.5 min, 72°C for 1.5 min, 72°C for 10 min
OXA-48-R	5’-CACACAAATACGCGCTAACC-3’			
NDM-F	5’-CACCTCATGTTTGAATTCGCC-3’	948bp	(1) Enzyme activation; (2) 30 cycles of initial denaturation annealing extension; (3) final extension	95°C for 15 min, 94°C for 30s, 55°C for 40s, 72°C for 1 min, 72°C for 10 min
NDM-R	5’-CTCTGTCACATCGAAATCGC-3’			

Correlation of carbapenemase genes with phenotypic carbapenem resistance

The CRKP isolates were subjected to a modified carbapenem inactivation method (mCIM) for detection of Ambler class A and B and OXA-48-like carbapenemases and if found positive were further subjected to EDTA-modified carbapenem inactivation method (eCIM). Isolates with both mCIM and eCIM tests positive were labeled as producing metallo-β-lactamases and those with positive mCIM but negative eCIM test were labeled as producing serine carbapenemases.

To know the spectrum of antibiotic resistance of the CRKP isolates, an antibiotic susceptibility assay by Kirby-Bauer disk diffusion method was performed. For colistin, minimum inhibitory concentrations (MICs) were determined by the broth microdilution method for which the breakpoint of <2 mg/ml was considered senstive. All the tests were performed and interpreted as per Clinical and Laboratory Standard Institute (CLSI) 2018 guidelines [9].

Clinical correlates

Clinical details regarding demographic data, history of previous hospitalization due to a medical and/or surgical illness, and any invasive procedure during the ICU stay of the patients were collected.

Statistical analysis

All the statistical analysis was done using MedCalc online software (https://www.medcalc.org/calc/odds_ratio.php) and GraphPad Prism Version 7 (San Diego, CA: GraphPad Software; www.graphpad.com). Statistical significance for parametric and non-parametric variables between the two carbapenemase genes was tested by chi-square and Fisher’s exact test and p-values <0.05 were considered as significant. Ninety-five percent confidence intervals (CIs) were calculated for various outcomes among the isolates producing the two genes to know the possible association between the presence of a gene and an outcome.

## Results

A total of 49 of 90 *K. pneumoniae* (54.4%) isolates gave a positive result for one or more carbapenemase genes, which were labeled as CRKP. Of these, 30 (61.2%) were positive for blaNDM gene only, 13 (26.5%) were positive for the blaOXA48-like gene only, and six (12.2%) were positive for both.

Laboratory correlates

A total of 38 isolates were positive for mCIM phenotypic test of which 28 also gave a positive eCIM test. Hence these 28 isolates were labeled as harboring metallo-β-lactamases and the remaining 10 as producing serine carbapenemases. A total of 11 isolates that were positive for carbapenem-resistant genes (five for blaOXA48-like gene only and six for both) were negative for mCIM test. Hence considering PCR as the gold standard, the sensitivity and specificity of phenotypic mCIM test were found to be 77.6% (95% CI = 63.4-88.2%) and 100% (95% CI = 91.4-100%), respectively. Positive and negative predictive values were 100% and 78.9%, respectively, and the overall accuracy of mCIM was calculated as 87.8% (95% CI = 79.2-93.7%).

All the 49 CRKP isolates were resistant to ertapenem, cephalosporins, and aztreonam. Isolates with only blaNDM gene were significantly more susceptible to gentamicin (p = 0.003), tobramycin (p = 0.039), ciprofloxacin (p = 0.008) and amoxicillin/ clavulanic acid (p = 0.016). In contrast, those with blaOXA48-like gene only were significantly susceptible to trimethoprim-sulphamethoxazole (p = 0.0003). Colistin and polymyxin B resistance was not found in any isolate. However, the presence of OXA-48 gene was significantly associated with increased MIC50 of colistin (2mg/ml) (p = 0.02, chi-square = 5.305) (Table 2).

**Table 2:** Antibiotic resistance pattern of isolates harboring the two carbapenemase genes OXA-48: oxacillinases β-lactamases; NDM: New Delhi metallo-β-lactamase

Name of antibiotic	Phenotypic resistance in isolates with blaNDM gene only (%) (n = 30)	Phenotypic resistance in isolates with bla OXA48-like gene only (%) (n = 13)	p- Value	Phenotypic resistance in isolates with both the genes n (%)
Ertapenem (10µg)	30 (100)	13 (100)	-	6 (100)
Aztreonam (30µg)	30 (100)	13 (100)	-	6 (100)
Amikacin (30µg)	23 (76.7)	13 (100)	0.08	6 (100)
Gentamycin (10µg)	16 (53.3)	13 (100)	0.003	6 (100)
Tobramycin (10µg)	21 (70)	13 (100)	0.039	6 (100)
Ciprofloxacin (5µg)	18 (60)	13 (100)	0.008	6 (100)
Levofloxacin (5µg)	16 (53.3)	13 (100)	0.135	6 (100)
Cefazolin (30µg)	30 (100)	13 (100)	-	6 (100)
Cefepime (30µg)	30 (100)	13 (100)	-	6 (100)
Ceftriaxone (30µg)	30 (100)	13 (100)	-	6 (100)
Amoxycillin-clavulanate (20/10µg)	22 (73.3)	4 (30.8)	0.016	6 (100)
Piperacillin-tazobactam (100/10µg)	28 (93.3)	13 (100)	1.00	6 (100)
Trimethoprim and sulphamethoxazole	30 (100)	7 (53.8)	0.0003	6 (100)
Colistin (minimum inhibitory concentration)	0 (0)	0 (0)	-	0 (0)

Clinical correlates

These 49 CRKP isolates were obtained from blood samples of 21 (42.9%) patients from pediatric ICUs and 28 (57.1%) patients from adult ICUs. NDM was more commonly observed in the pediatric population but OXA-48 was more common in adults. Case fatality, associated comorbidities, and empiric carbapenemase treatment were more commonly seen in patients with OXA-48, though the difference was not statistically significant. It should be noted that case fatality occurred in all six patients who were infected with CRKP harboring both genes (Table 3).

**Table 3:** Association of outcome and carbapenemase genes OXA-48: oxacillinases β-lactamases; NDM: New Delhi metallo-β-lactamase

Parameters	Outcome in patients with blaNDM gene only (%) (n = 30)	Outcome in patients with blaOXA48-like gene only (%) (n = 13)	p- Value	Outcome in patients with both the genes n (%)
Pediatric population (<18 years)	13 (43.3)	4 (30.8)	0.51	4 (66.7)
Adult population (18-70 years)	17 (56.7)	9 (69.2)	0.51	2 (33.3)
Mortality	16 (53.3)	9 (69.2)	0.50	6 (100)
Any associated comorbidity (chronic renal disease/diabetes mellitus, malignancy/others)	19 (63.3)	9 (69.2)	1	5 (83.3)
Empiric carbapenemase treatment	8 (26.7)	7 (53.8)	0.16	2 (33.3)

## Discussion

Infection with carbapenem-resistant *K. pneumoniae* is a dreaded complication associated with several hospital-associated risk factors. The proportion of CRKP isolates among invasive *K. pneumoniae* infections has been estimated to vary in different geographical regions. In India, the rates of carbapenem resistance have seen a sharp increase of 35% in just two years (9% in 2008 to 44% in 2010). Likewise, the rates in Italy soared to 60% in 2013 from undetectable levels in 2008 [9].

The carbapenemases have been classified into various groups as per Ambler classification. A multicentric retrospective study from India demonstrated that of the 344 retrospective *K. pneumoniae* isolates from eight centers across India, blaOXA48-like predominated (73.2%) followed by blaNDM-1/5 (24.4%) [5]. But in several other studies and in our study, NDM was found to be the major cause of carbapenem resistance in India followed by the OXA-48-like group [10,11]. The present study also showed that in our hospital setting NDM is the most common carbapenemase. Additionally, corroborating the present study, co-production of NDM-1 with other carbapenemases has been detected amongst Enterobacteriaceae isolates in many parts of India [12,13]. Looking at the variable data, it becomes mandatory for all the hospitals to continuously monitor the prevalent carbapenemases and highlight the noticed changing trend.

NDM, first detected in 2009, is epicentral to India but has now spread internationally. NDM-producing CRKP is known to be associated with high morbidity and mortality worldwide [14]. OXA-48 was first identified in Istanbul, Turkey, from a CRKP isolate. Owing to the usual location of NDM and OXA-48 on plasmid and transposon (Tn1999) respectively, the genes are easily transferable and can therefore spread easily [15]. Therefore, for infection prevention and control in a hospital setting, surveillance of these genes is imperative.

Studies have reported a sensitivity and specificity of mCIM ranging between 96.1-99% and 92.9-100%, respectively, for detection of CRKP as compared to the molecular tests [16,17]. In contrast, in the present study, the phenotypic mCIM test was found to have only moderate sensitivity (77.6%) and negative predictive value (78.9%) as compared to the conventional PCR, and all the phenotypically missed cases produced OXA-48 type carbapenemases singly or in combination with NDM. It should be noticed that the original CIM test was also found to give very promising results initially, with high sensitivity for detection of different types of carbapenemases and excellent sensitivity. Later, lower sensitivities for OXA-48 type carbapenemases varying from 50% to 91% were reported [18]. It is already known that it is difficult to identify this class of carbapenemases using phenotypic tests because they can result in low carbapenem MICs [19]. Hence, mCIM test should be verified for Indian strains producing OXA-48 type carbapenemases. Besides, other mechanisms such as altered membrane permeability, increased efflux pumps, or hyperproduction of extended-spectrum β-lactamases (ESBLs) or AmpC β-lactamases (cephalosporinases) may also lead to carbapenem resistance [19]. Hence, relying only on phenotypic tests like mCIM for the detection of carbapenemase detection may at times be misleading. Though mechanisms of carbapenem resistance may not alter the clinical outcome, such information is essential for infection control purposes because carbapenemase producers spread more easily.

We could not find any statistical association between mortality rates and the type of carbapenemase. A meta-analysis reported a pooled mortality of 42.1% in patients infected with CRKP compared to 21.2% in those infected with carbapenem-sensitive *K. pneumoniae*. When mortality between types of carbapenemases was studied, no statistical difference was observed (47.7% in patients infected with *K. pneumoniae* carbapenemase (KPC)-producing *K. pneumoniae* versus 46.7% in those infected with VIM-producing *K. pneumoniae*) [20]. However, in the present study, mortality occurred in all the patients with *K. pneumoniae* isolates producing both NDM and OXA-48. The high mortality rates in patients infected with CRKP isolates are attributed to the lack of appropriate antibiotics combined with underlying patient comorbidities.

In the present study, it was found that isolates with only blaNDM gene were significantly more susceptible to aminoglycosides and ciprofloxacin and those with blaOXA48-like gene only were significantly more susceptible to trimethoprim-sulphamethoxazole. This is in contrast to a previous study that postulated that OXA-48 producers remain susceptible to gentamicin, while this is rare for NDM [21]. Generally, it is said that NDM producers remain sensitive to aztreonam and OXA-48 producers test sensitive to extended-spectrum cephalosporins in about 20% of the cases [22]. Owing to these contrasting results possibly due to the simultaneous existence of multiple resistance mechanisms, optimal treatment of infections with CRKP is currently not known, and based on the laboratory’s AST report; the treating physician needs to tailor individual patient therapy. It was observed that all the CRKP isolates did not have raised MICs for different carbapenems and some isolates were even sensitive to imipenem. These variations in the MIC values for different carbapenems may be influenced by the simultaneous presence of other resistance mechanisms. Therefore, combination therapy of carbapenems with other drugs such as colistin, polymyxins, tigecycline, gentamicin, or fosfomycin may be tried. The MIC values of colistin were found to be within the effective range for all the isolates possessing the carbapenemase genes, though the presence of the OXA-48 gene was significantly associated with increased MIC50 (2mg/ml) of colistin (p = 0.02). These drugs are used as the last line of drugs for the treatment of carbapenemase-producing isolates. Increased MIC levels are a threat and colistin resistance has already been reported in such isolates [23]. The biggest flaws of this study were the small sample size and single-center study; larger sample size with multicentric analysis is required to validate the results.

## Conclusions

The situation is scary, as it would mean going back to the pre-antibiotic era. Some newer drug combinations like ceftazidime-avibactam are under evaluation for the treatment of CRKP; however, currently, their treatment remains elusive. To conclude, no significant difference in mortality was observed in NDM and OXA-48-producing CRKP isolates, but 100% mortality was observed with isolates producing both the carbapenemases. Increased MIC levels of colistin were observed, though they lay in the sensitive range. Statistically, a significant difference was observed in the susceptibility of various antibiotics in isolates producing NDM and OXA-48 carbapenemases.
